# Editorial: Molecular epidemiology and phylogeny of tick-borne pathogens in ixodid ticks and vertebrate hosts

**DOI:** 10.3389/fvets.2024.1464982

**Published:** 2024-09-13

**Authors:** Munir Aktas, Kursat Altay

**Affiliations:** ^1^Department of Parasitology, Faculty of Veterinary Medicine, Firat University, Elazig, Türkiye; ^2^Department of Parasitology, Faculty of Veterinary Medicine, Sivas Cumhuriyet University, Sivas, Türkiye

**Keywords:** ixodid ticks, tick-borne diseases, molecular detection, epidemiology, phylogeny

The impact of ticks and tick-borne diseases on human and animal health continues to increase almost all over the world. The factors such as global warming, human and animal movements between countries and even continents, and deforestation strengthen this effect (1, 2). The interest of the scientific community in these vectors and the diseases, which are important for human and animal health as well as animal breeding [vectors and vector-borne diseases may cause economic losses, approximately US$22–30 billion per year, among cattle herds (3)] has always been high. Intensive studies have been carried out at microscopic, serological, and molecular levels to determine the etiology and epidemiology of these diseases. Microscopic methods were preferred in these studies due to their low cost, rapid results, and easy application, but they were insufficient in identifying carrier animals or making species identifications (4, 5). Serological tests such as indirect fluorescent antibody test (IFAT), complement fixation test (CFT), and enzyme-linked immunosorbent assay (ELISA) are especially useful in epidemiological studies, but they can give false positive results due to cross antigenic reactions and negative results in cases of low immunity (4, 6). In recent years, molecular diagnostic methods including polymerase chain reaction, reverse line blotting, DNA sequencing and phylogeny have become the preferred methods all over the world for the identification and epidemiology of these diseases. While these methods stand out with their high sensitivity and specificity, they enable the identification of new genotypes, strains, and even species or their hosts. The primary application of molecular tools highlights the specific and precise description of tick-borne pathogens to solve many taxonomic problems. In recent years, the application of molecular diagnostic tools, increasingly used for the identification and taxonomy of tick-borne pathogens, has provided new insight and knowledge with regards to molecular epidemiology and phylogeny. Understanding of tick-parasite-host interactions, transmission dynamics, vector biology, and ecology are essential to develop effective control measures (1).

The Research Topic titled “*Molecular epidemiology and phylogeny of tick-borne pathogens in ixodid ticks and vertebrate hosts*” has been intended to bring together current studies on the molecular epidemiology of ticks and tick-borne diseases, ticks, parasite, and host interaction, molecular taxonomy of pathogens, genetic differences and vector potential of ticks. For this purpose, important studies on bacterial and parasitic pathogens found in different hosts and ticks have been published after scientific evaluations.

Mahdy et al. investigated piroplasma infections in 531 camels in Egypt using conventional and molecular methods. The researchers combined blood-stain with Di?-3 stain, uniplex PCR (uPCR), multiplex PCR (mPCR), and amplicon sequencing methods together. In the study, 58 out of 531 camel samples were found to be infected with Di?-3 stain, whereas the pathogen DNA was detected in 203 samples using PCR. They determined the presence and prevalence of *Theileria equi, Babesia caballi, B. bigemina, B. bovis, B. vulpes, Babesia* sp., and *Theileria* sp. infections in this host. With this study, it was predicted that the combined use of molecular methods could have advantages in the identification of species, and it was also revealed that molecular methods enable the identification of different species in different hosts.

Aljasham et al. investigated the role of hard ticks (*Hyalomma dromedarii*) as reservoirs for antimicrobial resistant (AMR) bacteria for the first time. They have identified different AMR bacteria (*Staphylococcus, Enterococcus, Streptococcus, Klebsiella Pseudomonas*, and *Stenotrophomonas*) some of which have pathogenic potential (*Enterococcus, Streptococcus, Pseudomonas*, and *Klebsiella*) to humans and animals. In the study, it was determined that bacterial isolates obtained from ticks were resistant to one or more of 9 antimicrobial agents [pipracillins (ampicillin, amoxicillin/clavulanic acid, piperacillin/tazobactam, benzylpenicillin, oxacillin), cephalosporin (cephalothin, cefoxitin, ceftazidime, ceftriaxone, cefepime), tetracyclines (tetracycline, tigecycline), glycopeptide (teicoplanin, vancomycin), macrolides (erythromycin), lincomycin (clindamycin), rifamycin (rifampicin), nitrofuran (nitrofurantoin), and, sulfonamides (trimethoprim/sulfamethoxazole)]. They also showed that *Staphylococcus aureus* may be part of the tick microbiota. Although the data obtained from the study emphasized that AMR bacteria may circulate with ticks, further studies are needed to determine the role of ticks in the circulation of these bacteria between humans and animals.

Khan et al. researched *Borrelia* spp. in hard ticks collected from domestic animals in Khyber Pakhtunkhwa, Pakistan. The researchers collected 729 tick species belonging to 16 species from 264 domestic animals (goat, sheep, cattle, camel, horse, and dog) and performed DNA extraction after creating tick pools from these tick species. The DNAs obtained were investigated with primers amplifying the *16S rRNA* and *flaB* gene regions of *Borrelia* species and positive samples were subjected to DNA sequence analysis. Phylogenetic analysis of the nucleotide sequences obtained from DNA sequence analysis was performed and the presence of *Borrelia theileri* and *Borrelia* sp. was demonstrated. *Borrelia theileri* was detected in *Rhipicephalus microplus* and *Rhipicephalus turanicus*, while *Borrelia* sp was detected in *Haemaphysalis cornupunctata* and *Haemaphysalis sulcata* ticks collected from goats. This study contributes to expanding the geographical and tick host range of RF borreliae, which could support future research efforts focused on veterinary health.

Nehra et al. investigated the *Theileria equi* genotypes that are transmitted by ticks and can cause significant economic losses in the world, especially by infecting horses and other single-hoofed animals. The researchers investigated the *T. equi* genotypes circulating in the world by phylogenetic analysis of the nucleotide sequences belonging to the V4 hypervariable region of the *18S rRNA* gene of *T. equi*, which were identified in different regions of the world and uploaded to GenBank. The researchers selected 736 *T. equi* sequences containing the *18S rRNA* gene of *T. equi* from 14,792 nucleotide sequences in GenBank and the phylogenetic features of these sequences were examined using various online programs. Phylogenetic analyses revealed that these 736 sequences belong to clade A (n:203), B (n:389), C (n:78), and D (n:72). In addition, phylogenetic analyses also showed that high nucleotide differentiations are present in the V4 region, especially nucleotide positions between 113 and 183 on the gene. The study showed that Clade A circulates in 31 countries in the continents Asia, Africa, Europe, North America and South America, Clade B in 21 countries in the continents Asia, Africa, and Europe, Clade C in 13 countries in the continents Asia, Africa, Europe, North America, and South America, and Clade D in 12 countries in the continents Asia, Africa, and Europe. In summary, with this study title, important studies including up-to-date data on *Theileria* and *Babesia* species circulating in camels in Egypt, antibiotic-resistant bacteria found in ticks, ticks collected from goats, sheep, cattle, camels, horses, and dogs in Pakistan, and bacterial pathogens found in these ticks, genetic characteristics of *T. equi* circulating in different regions of the world and geographical distribution of these strains have been published and contributed to the scientific community.
